# Supplementary material for: Child-Sensitive WASH Composite Score and the Nutritional Status in Cambodian Children
Source: Nutrients. 2019 Sep 7;11(9):2142. doi: 10.3390/nu11092142 (PMC6770572; doi:10.3390/nu11092142)
Supplement: Supplementary file 1 [file nutrients-11-02142-s001.pdf]

Supplementary Table 1. Sub-score classification. Variables used to create JMP-CS and National-CS water, sanitation and hygiene sub-scores which values were classified as “improved” or “not improved”.

	Water Sub-Score			
	Improved		Not Improved	
**JMP-CS**	**Baseline**	**Follow-Ups**	**Baseline**	**Follow-Ups**
Main source of water	Piped into dwelling<br>Piped into yard/plot<br>Tube well or borehole<br>Pumping well<br>Rainwater<br>Tanker truck or water vendor<br>Bottled water	Piped into dwelling<br>Piped into yard/plot<br>Public tap/standpipe<br>Tube well or borehole<br>Protected dug well<br>Rainwater<br>Protected spring<br>Bottled water<br>Tanker truck or water vendor	Dug well<br>River/dam/stream/lake/pond/ canal/irrigation channel<br>Don’t know	Unprotected dug well<br>River/dam/stream/lake/pond/ canal/irrigation channel<br>Unprotected spring<br>Don’t know
Location of water source	In own dwelling<br>In own yard/plot	In own dwelling<br>In own yard/plot	Shared with neighbour<br>Public place<br>Don’t know	Elsewhere<br>Don’t know
**National-CS**				
Main source of water	Piped into dwelling<br>Piped into yard/plot<br>Tube well or borehole<br>Pumping well<br>Rainwater	Piped into dwelling<br>Piped into yard/plot<br>Public tap/standpipe<br>Tube well or borehole<br>Protected dug well<br>Rainwater<br>Protected spring	Dug well<br>River/dam/stream/lake/pond/ canal/irrigation channel<br>Tanker truck or water vendor<br>Bottled water<br>Don’t know	Unprotected dug well<br>River/dam/stream/lake/pond/ canal/irrigation channel<br>Unprotected spring<br>Bottled water<br>Tanker truck or water vendor<br>Don’t know
Location of water source	In own dwelling<br>In own yard/plot	In own dwelling<br>In own yard/plot	Shared with neighbour<br>Public place<br>Don’t know	Elsewhere<br>Don’t know
Frequency of water treatment	Yes	Yes always	No<br>Sometimes<br>Don’t know	No<br>Yes sometimes<br>Don’t know

Method used to treat the water	Boil<br>Bleach<br>Stain<br>Water filter<br>Solar	Boil<br>Bleach chlorine<br>Strain through a cloth<br>Ceramic filter<br>Solar	With Alum<br>Stand and settle<br>Don't know	Stand and settle<br>Don't know
Frequency of child's water treatment	Yes	Yes always	No<br>Sometimes<br>Don't know	No<br>Yes sometimes<br>Don't know
	**Sanitation Sub-Score**			
	**Improved**		**Not improved**	
**JMP-CS**	**Baseline**	**Follow-ups**	**Baseline**	**Follow-ups**
Type of toilet	In-house<br>Outhouse	Flush/pour flush toilet flush to piped sewer system<br>Flush/pour flush toilet flush to septic tank<br>Flush/pour flush toilet flush to pit latrine<br>Flush/pour flush toilet flush to somewhere else<br>Flush/pour flush toilet flush don't know where<br>Ventilated improved pit latrine<br>Pit latrine with slab<br>Composting toilet	Share<br>Public<br>Dry<br>Field/forest<br>Refuse to respond<br>Don't know	Pit latrine without slab/open pit<br>Bucket toilet<br>Hanging toilet<br>Field/forest<br>Refuse to respond<br>Don't know
Strategy used to dispose child excreta	Child used toilet/latrine<br>Thrown by caretaker in toilet/latrine<br>Buried	Child used toilet/latrine<br>Thrown by caretaker in toilet/latrine<br>Buried	Thrown into drain or ditch<br>Thrown into garbage<br>Left in open<br>Refuse to respond<br>Don't know	Thrown into drain or ditch<br>Thrown into garbage<br>Left in open<br>Refuse to respond<br>Don't know
**National-CS**				
Type of toilet	In-house<br>Outhouse	Flush/pour flush toilet flush to piped sewer system<br>Flush/pour flush toilet flush to septic tank	Share<br>Public<br>Dry<br>Field forest	Pit latrine without slab/open pit<br>Bucket toilet<br>Hanging toilet

		Flush/pour flush toilet flush to pit latrine<br>Flush/pour flush toilet flush to somewhere else<br>Flush/pour flush toilet flush don't know where<br>Ventilated improved pit latrine<br>Pit latrine with slab<br>Composting toilet	Refuse to respond<br>Don't know	Field/forest<br>Refuse to respond<br>Don't know
Strategy used to dispose child excreta	Child used toilet/latrine<br>Thrown by caretaker in toilet/latrine	Child used toilet/latrine<br>Thrown by caretaker in toilet/latrine	Thrown into drain or ditch<br>Thrown into garbage<br>Buried<br>Left in open<br>Refuse to respond<br>Don't know	Thrown into drain or ditch<br>Thrown into garbage<br>Buried<br>Left in open<br>Refuse to respond<br>Don't know
		**Hygiene Sub-Score**		
	**Improved**		**Not improved**	
**JMP-CS**	**Baseline**	**Follow-ups**	**Baseline**	**Follow-ups**
Washes hands after cleaning the child's bottom and/or after moving faeces	Yes, after cleaning the child,<br>Yes, after moving the faeces	Yes, after cleaning the child<br>Yes, after moving the faeces	No	No
**National-CS**<br>*Washes hands after cleaning the child's bottom and/or after moving faeces*	Yes, after cleaning the child,<br>Yes, after moving the faeces	Yes, after cleaning the child<br>Yes, after moving the faeces	No	No
